# Estimating snakebite incidence from mathematical models: A test in Costa Rica

**DOI:** 10.1371/journal.pntd.0007914

**Published:** 2019-12-02

**Authors:** Carlos A. Bravo-Vega, Juan M. Cordovez, Camila Renjifo-Ibáñez, Mauricio Santos-Vega, Mahmood Sasa

**Affiliations:** 1 Research Group in Mathematical and Computational Biology (BIOMAC), Department of biomedical engineering, University of los Andes, Bogotá, Colombia; 2 Corporación colombiana de investigación agropecuaria, AGROSAVIA, Bogotá, Colombia; 3 Instituto Clodomiro Picado and Escuela de Biología, Universidad de Costa Rica, San José, Costa Rica; Baylor College of Medicine, UNITED STATES

## Abstract

**Background:**

Snakebite envenoming is a neglected public health challenge that affects mostly economically deprived communities who inhabit tropical regions. In these regions, snakebite incidence data is not always reliable, and access to health care is scare and heterogeneous. Thus, addressing the problem of snakebite effectively requires an understanding of how spatial heterogeneity in snakebite is associated with human demographics and snakes’ distribution. Here, we use a mathematical model to address the determinants of spatial heterogeneity in snakebite and we estimate snakebite incidence in a tropical country such as Costa Rica.

**Methods and findings:**

We combined a mathematical model that follows the law of mass action, where the incidence is proportional to the exposed human population and the venomous snake population, with a spatiotemporal dataset of snakebite incidence (Data from year 1990 to 2007 for 193 districts) in Costa Rica. This country harbors one of the most dangerous venomous snakes, which is the Terciopelo (*Bothrops asper*, Garman, 1884). We estimated *B*. *asper* distribution using a maximum entropy algorithm, and its abundance was estimated based on field data. Then, the model was adjusted to the data using a lineal regression with the reported incidence. We found a significant positive correlation (R^2^ = 0.66, p-value < 0.01) between our estimation and the reported incidence, suggesting the model has a good performance in estimating snakebite incidence.

**Conclusions:**

Our model underscores the importance of the synergistic effect of exposed population size and snake abundance on snakebite incidence. By combining information from venomous snakes’ natural history with census data from rural populations, we were able to estimate snakebite incidence in Costa Rica. The model was able to fit the incidence data at fine administrative scale (district level), which is fundamental for the implementation and planning of management strategies oriented to reduce snakebite burden.

## Author summary

Snakebite envenoming is an important health issue that affects mostly rural populations in tropical countries. Antivenom is the only effective treatment, but it is not always available in every medical center located in rural areas. Additionally, the number of snakebite cases is usually not known, or severely underestimated for several tropical countries. As a consequence, antivenom distribution and disease management are hard to optimize, then the estimation of snakebite incidence has been proposed as a key factor to improve snakebite management. To the date, studies have used statistics and community-based surveys to estimate incidence, but these approaches are still challenging. Here our goal is to develop a mathematical model that can estimate the geographic patterns of snakebite incidence. By applying a simple model, which is based on the law of mass action, we successfully captured the spatial variation of snakebite incidence in Costa Rica. The inputs for this model are venomous snakes’ census and presence records, and human population data. Our work gives the basis for the development of more complex models based on the law of mass action to estimate several epidemiological variables associated with snakebite in rural populations.

## Introduction

Snakebite envenoming is mainly a disease of rural populations that affects up to 2.7 million people and kills as many as 95,000 worldwide per year [1–4]. The highest snakebite mortality rates occur in tropical countries with the lowest per capita government expenditure on health and the lowest per capita GDP [5,6]. The public health challenge of this Neglected Tropical Disease (NTD) is that the most afflicted communities are the least able to access or afford effective antivenom treatment. The development of an appropriate response to snakebite requires a reasonable estimation of its burden at local and regional scales, and the understanding of the drivers of its spatiotemporal patterns. Understanding these elements could contribute to: 1) public health planning by government authorities, 2) optimization of production and distribution of antivenom, 3) the design of control strategies to minimize snakebite incidence, and 4) correct training of the medical staff who will treat envenomed patients [7]. However, as it happens with other NTDs, epidemiological data of snakebite incidence is not readily available for several countries, especially in Tropical regions [8,9].

The most cost-effective way to obtain reliable NTDs epidemiological data is via medical records, especially in countries where these reports are mandatory [10]. However, many governments still do not require health centers to inform of snakebite cases, making epidemiological data scarce or incomplete [2]. In addition, countries with large rural and indigenous populations tend to exhibit an underestimation in their records due to population disbelief in modern medicine and a limited access to health care [2,10,11]. For example, in West Bengal, a community-based study using door-to-door surveys estimated that only 22% of snakebite victims sought medical attention, and that medical centers only reported 7% of the total cases [12]. Likewise, another community-based study performed in eastern Nepal estimated a total of 4078 snakebites, but the whole country only reported 480 cases for the same year (89% of the cases were not reported) [13]. However, such studies require numerous interviewers and resources that are not readily available in most instances. Therefore the improvement of the surveillance systems and the estimation of snakebite burden is central for the control and management of this NTD [8].

Statistical inference has emerged as an effective tool for estimating snakebite burden without detailed epidemiological surveys. For example, several meta-analyses have built linear statistical models to infer epidemiological parameters in countries where data is not available [2,6,14]. Although estimations of snakebite incidence and mortality can also be made with this approach, they only provide broad approximations based on inferences, and do not take into account the processes underlining human-snake interactions [15]. Compartmental epidemiological models, another tool for estimating epidemiological variables, are based on the interactions between populations involved in disease’ epidemiology [15,16]. These models have been used widely for several NTDs such as Chagas disease, Dengue and Malaria, but not for snakebite [17,18]. One of the main assumptions in these models is the law of mass action, namely: the encounters between two populations that are moving randomly are proportional to the multiplication of the abundance of both populations [19]. Using the law of mass action, the incidence for several diseases can be defined as proportional to the total encounters between an infectious population and a susceptible population [15].

**Fig 1:**
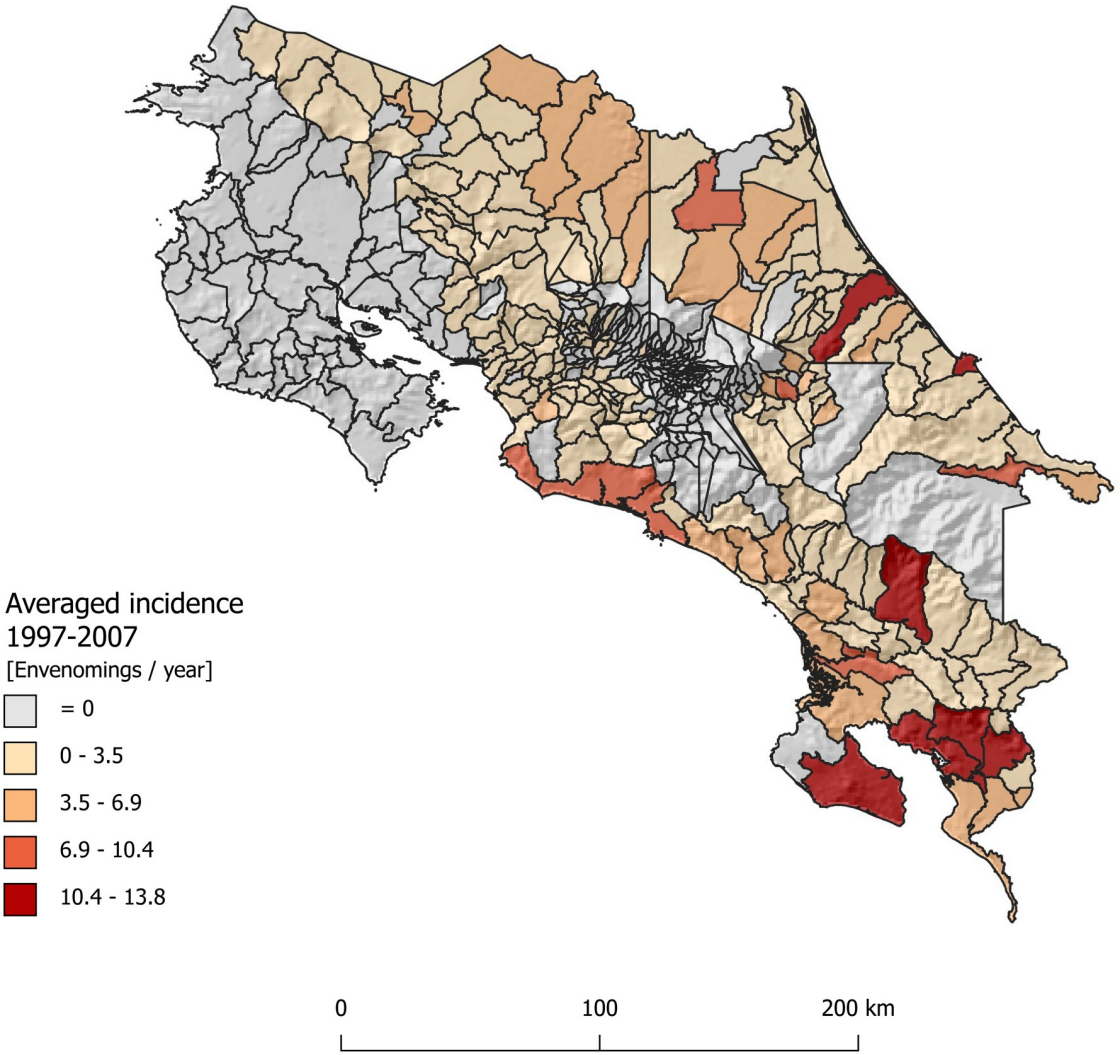
Study area and reported snakebite incidence per district, 1990–2007. Costa Rica is a tropical country in the Central American isthmus. The incidence is shown for the districts where the study species (*Bothrops asper*) is present. We supposed that in these districts the study species was responsible for all snakebites. Note that highlands and dry northwest plains have no reported incidence of snakebites attributable to this species, whereas the wet lowlands in the central and South Pacific regions have the highest incidence. (Map was produced by using QGIS Geographic Information System, Open Source Geospatial Foundation Project. http://qgis.osgeo.org).

As an effort to evaluate the possible use of compartmental epidemiological models in the prediction of snakebite incidence, we formulated a model based on the law of mass action to estimate the snakebite burden in Costa Rica. We selected this country because of its highly reliable snakebite epidemiological data, that allows final evaluation of our model estimations (Fig 1) [14]. To do so, we predicted the distribution of *B*. *asper* in the country using niche modeling based on collection records. This species is considered the most medically important species of venomous snakes in the country and it is responsible for over 70% of the snakebites [20,21] (see below). Then, we conducted fieldwork to assess *B*. *asper* relative abundance and to calibrate the spatial model. Finally, we evaluated the performance of the model capturing the spatial variation of reported snakebite incidence.

## Materials and methods

### Study area

Costa Rica (Fig 1) is a country with a reliable snakebite dataset, where the public health system incorporates medical centers throughout its territory and a well-established social security system [22]. In this country, snakebite reporting is mandatory, and statistics on snake envenomation are consistent [23]. In addition, Costa Rica is home to the Instituto Clodomiro Picado at the University of Costa Rica, a research center and antivenom production facility that ensures that antivenom is accessible and available throughout the national territory. All these characteristics make Costa Rica a great country to asses, parameterize, and evaluate a mathematical model to predict snakebite incidence.

### Applying the law of mass action to predict snakebite

The law of mass action states that the number of encounters between two populations are proportional to the multiplication of their sizes [15]. We can apply this law to snakebite, where the total encounters between venomous snakes and exposed humans will be proportional to the size of each population. Then, if we multiply these encounters by the conditional probability that an encounter ends in a bite, we can estimate snakebite incidence:

$$I_{i}=\theta \times \beta \times S_{i}\times V_{i}$$ (1)

Where the subindex *i* denotes the administrative unit in which the model will be applied, *θ* is the conditional probability that snakebite occurs during an encounter, *β* is the contact rate between susceptible humans (*S*_*i*_) and venomous snakes (*V*_*i*_), and *I*_*i*_ is the predicted incidence.

One limitation of the model proposed in Eq 1 is quantifying the abundance of venomous snakes in the field (*V*_*i*_), which requires considerable sampling effort [24]. To overcome this limitation, we can multiply the contact rate between susceptible humans and venomous snakes (*β*) with the abundance of venomous snakes (*V*_*i*_) to get a new variable (*F*_*i*_) which represents the encounter frequency with venomous snakes per human per unit of time. This new variable is easier to estimate in the field because it only requires census of venomous snakes normalized by man-hour of sampling effort instead of absolute snake abundance. The proposed model incorporating the new variable is:

$$I_{i}=\alpha +\theta \times F_{i}\times S_{i}$$ (2)

Where the sub-index *i* denotes the administrative unit sampled–in this case districts, which are the finer administrative unit in Costa Rica, *α* is the intercept, *F*_*i*_ is the encounter frequency with venomous snakes, and *S*_*i*_ is the susceptible human population calculated from the reported rural population from 2000 census data (http://www.inec.go.cr/). The new parameter *α* allows the average of the regression residuals to be equal to zero [25]. Therefore, the *sum* of the predicted values will be equal to the *sum* of the observed values (i.e. The predicted *national* incidence will be equal to the reported *national* incidence). We used yearly snakebite incidence data from 1990 to 2007 reported by the Social Security of Costa Rica (Caja Costarricense del Seguro Social, CCSS), and we estimated the encounter frequency (*F*_*i*_) based on fieldwork.

### Estimation of encounter frequency with venomous snakes (*F*_*i*_)

**Fig 2:**
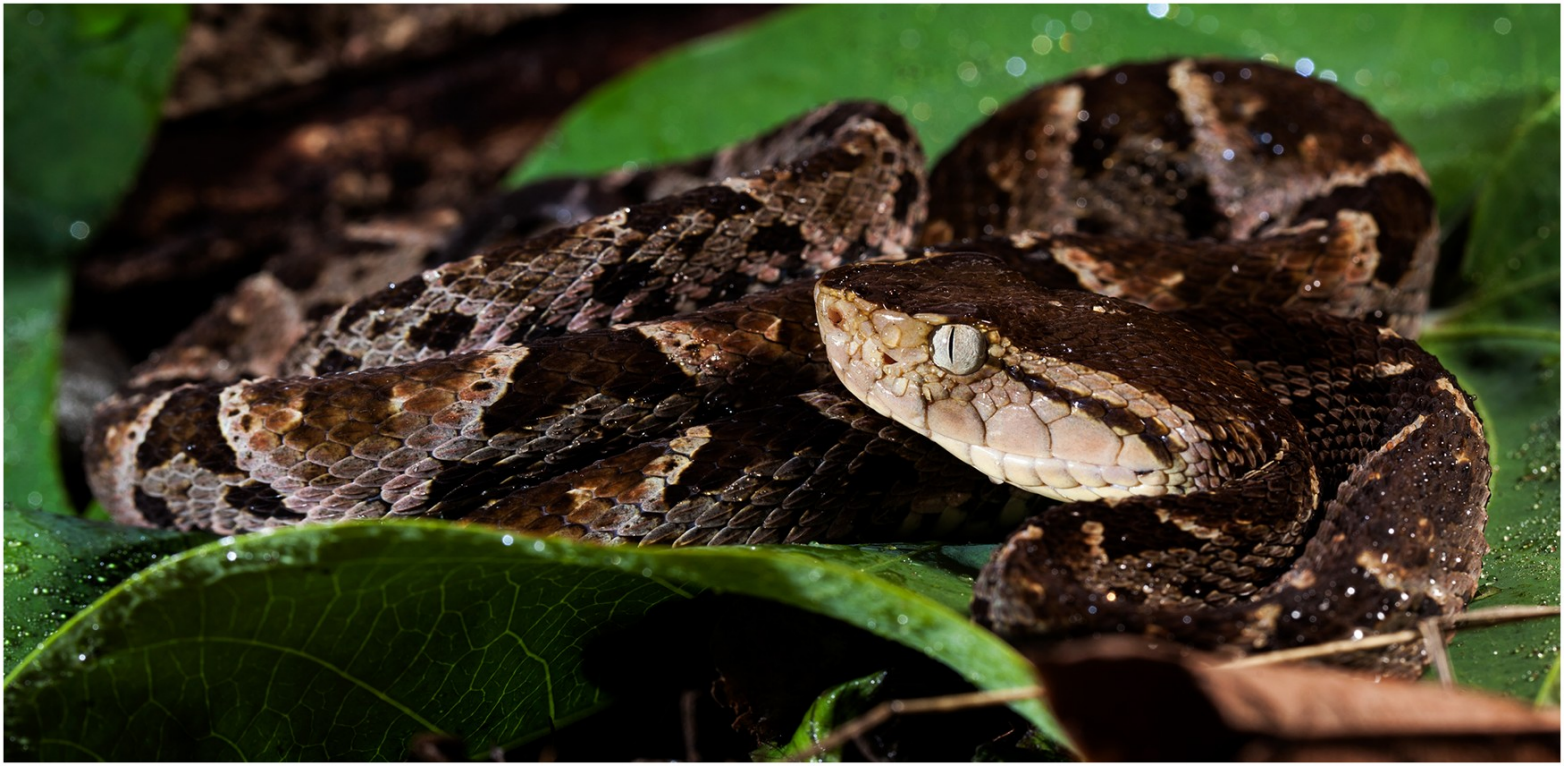
Study species: Terciopelo pit viper, *Bothrops asper*. This species is responsible for most snakebite envenomation throughout its distribution. It is distributed in humid lowlands from southern Mexico to northeastern South America. Photography was taken by Bravo-Vega CA.

Out of 146 species of snakes in Costa Rica, 24 are clinically relevant, including seven coral snakes (family Elapidae) and 17 pit vipers (family Viperidae) [26]. Among the pit vipers, the terciopelo (*Bothrops asper*, Fig 2) is responsible for more than 70% of snakebite cases in the region, due to its high relative abundance and its capacity to adapt to perturbed environments [20,21]. This species is distributed along the humid lowlands from Mexico and Central America to northern South America, where is commonly found in agricultural fields or in close proximity to human settlements [21,27]. We chose this species to test the proposed model because it causes most snakebites.

To estimate *F*_*i*_ (Eq 2), we established the distribution of *B*. *asper* in Costa Rica and then we determined the encounter frequency by searching for *B*. *asper* within its distribution (see description of fieldwork below).

### Distribution of *B*. *asper* in Costa Rica

To estimate the potential distribution of this species, we performed environmental niche modeling with Maxent (Maximum entropy algorithm [28]) because of its high performance when only occurrence data is available, and its ability to discriminate between suitable and unsuitable areas. We obtained *B*. *asper* presence data from locality records at the Instituto Clodomiro Picado, and we used environmental data from the Bioclim database on the WorldClim Server (http://www.worldclim.org/bioclim) [29]. The species distribution model was run using the dismo package in R [30,31]. To evaluate model precision, we computed a ROC (Receiver Operating Characteristic) curve based on 1000 random “pseudo-absence” background points and calculated true and false positive rates. With these rates, the area under the ROC curve (AUC) was computed, and finally the model performance was compared against a randomized prediction using the same environmental data [28].

To generate a presence/absence map based on Maxent results, we selected the threshold that has zero omission rate with the maximum predicted presence area [32], and we removed areas above 1200 m.a.s.l., the highest altitude limit reported for *B*. *asper* in Costa Rica [33]. The distribution maps were created using Quantum GIS (QGIS) software [34].

### Sampling encounter frequency (field work)

**Fig 3:**
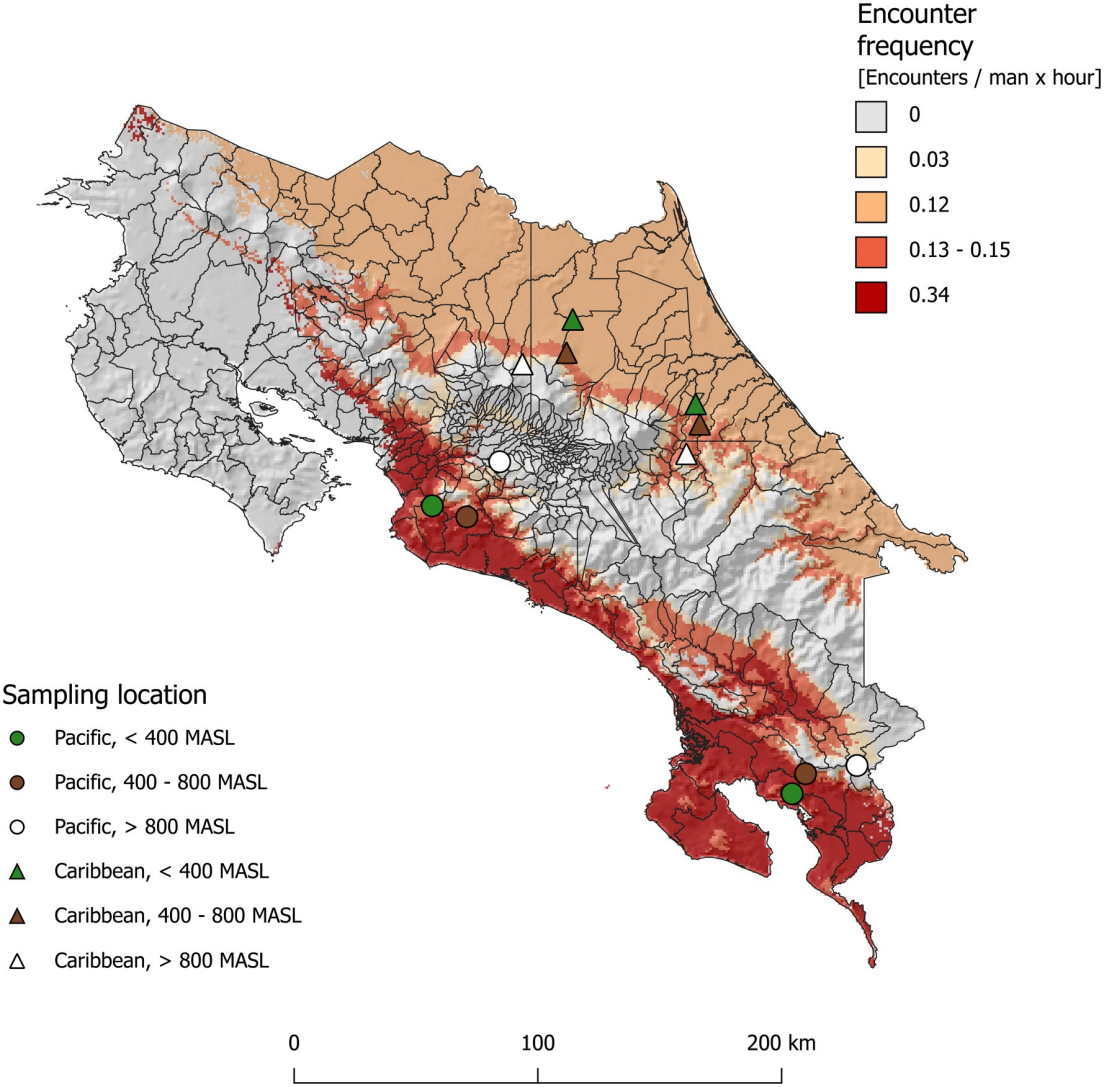
Sampling locations and predicted encounter frequency of *Bothrops asper* over its distribution. Each symbol corresponds with a sampling location. Circles are sampling locations in the Pacific versant, while triangles represent locations in the Caribbean versant. Green symbols are lowlands locations (Altitude below 400 m.a.s.l.), brown symbols are midlands locations (Altitude between 400 m.a.s.l and 800 m.a.s.l.), and white symbols are highlands locations (Altitude above 800 m.a.s.l.). Gray areas are places where the environmental niche model predicted absence for the study species. The other map colors show the value of the extrapolated encounter frequency. Encounter frequency was higher in the Pacific versant than in the Caribbean, and it tends to decrease with altitude. Map was produced by using QGIS Geographic Information System, Open Source Geospatial Foundation Project. http://qgis.osgeo.org.

A series of active search surveys for snakes were carried out during the dry season (January to April) and wet season (August to November) in 2017 at 12 sampling sites in Costa Rica, divided in six in the Pacific versant and six in the Caribbean versant (Fig 3). Sites were evenly distributed among three altitude ranges: 0–400 m, 400–800 m, and over 800 m. This selection was done because populations of *B*. *asper* varies among the two versants [35], and their abundance could decrease with elevation [21,36]. Additionally, with selected sampling sites we covered most of the ecoregions where *B*. *asper* is present in Costa Rica: The Caribbean zone, the north zone, the central pacific zone and the south pacific zone [37]. We only missed the Guatuso plain area. Given that these ecoregions are not severely heterogeneous, we assumed that the measured encounter frequency in selected sampling sites could be extrapolated throughout predicted distribution.

At each site, we sought for *B*. *asper* in the night during new moon periods across three land cover types: open perturbed areas (*i*.*e*. pastures or crops), closed canopy perturbed areas (*i*.*e*. plantain, oil palm or sugar cane crops), and unperturbed forest. The sampling effort was at least ten person-hours per land cover type per site. The number of observed individuals was recorded and normalized by man-hours of active searching, and then we extrapolated this frequency of encounters based on predicted distribution, altitude thresholds and versant. Finally, we computed the spatial-averaged encounter frequency per each district (*F*_*i*_).

### Model calibration

We assumed that *B*. *asper* was responsible for all snakebite cases because it has a very high incidence of envenomation. To calibrate our model, we performed linear regression between *F*_*i*_ × *S*_*i*_ (obtained as described above) and the reported incidence by the CCSS using a 99% confidence and prediction intervals. Our sample size consisted of 193 districts where snakebites were reported. The slope of the regression is the parameter *θ*, and the intercept is the parameter *α* (Eq 2). To evaluate the model, we computed r-squared using a 99% confidence level and we did a Pearson’s product moment correlation test between model residuals and predicted values.

To test model performance against different hypothesis, we compared it to four different models: ***i)*** A model with the intercept set equal to zero, ***ii)*** A linear model between the incidence and rural population, ***iii)*** A linear model between the incidence and the district-averaged encounter frequency, and ***iv)*** A multiple linear model of incidence as a function of rural population and the district-averaged encounter frequency. To compare the five models, we used r-squared and the Akaike information criterion (AIC). This criterion accounts for the trade-off between model prediction performance and the number of parameters, where the model with the lowest AIC will be the preferred one [38].

## Results

### Expected distribution of *B*. *asper* in Costa Rica

We estimated the presence of *B*. *asper* in 55% of the Costa Rican continental territory, encompassing mostly low and wet areas. The most important bioclimatic variables in the species distribution model (variables that account for the 80% of the variation of the prediction) were precipitation of driest quarter, precipitation of coldest quarter, minimum temperature of the coldest month, maximum temperature of the warmest month, precipitation of the wettest quarter, annual precipitation, annual mean temperature and mean temperature of the coldest quarter. The AUC value of the model is 0.79, which suggests that it is better at predicting presence points than a random model (AUC = 0.5), but is less than perfect (AUC = 1).

At field sites, we employed a total sampling effort of 562 person-hours, finding 186 snakes, of which 85 were *B*. *asper* (relative abundance of 0.45). Interestingly, the high relative abundance of 0.7 with respect to venomous snakes (Families *Elapidae* and *Viperidae*) supports the assumption that most snakebites are caused by *B*. *asper*. National averaged encounter frequency of *B*. *asper* is 0.15 vipers per person-hour, and it tends to decrease with elevation, with higher values in the southern Pacific lowlands (see map of extrapolated values in Fig 3).

### Modeling snakebite incidence in Costa Rica

An average of 438.5 snakebite cases were reported per year (minimum: 360 in 1991, maximum: 517 in 2006). Most cases occurred in the South Pacific and Caribbean lowlands and were distributed in elevations below 1400 m.a.s.l (Fig 1): These records could be attributed to *B*. *asper*. All models showed a significant p-value for the regression, but the proposed model (Eq 2, Model 1 in Table 1) was the best model (Highest r-squared and lowest AIC).

**Table 1:** Comparison between different models to estimate snakebite incidence.

Model	Structure	R -squared	AIC	Predicted national incidence (Cases/year)	Range for predicted national incidence (Cases/year)
Model 1*	*I*_*i*_ = *α* + *θ* × *F*_*i*_ × *S*_*i*_	0.665	741.44	438.5	361.29–515.82
Model 2	*I*_*i*_ = *θ* × *F*_*i*_ × *S*_*i*_	0.648	749.21	382.3**	344.99–419.68
Model 3	$I_{i}=\alpha +\hat{\theta }\times S_{i}$	0.344	871.3	438.5	313.38–563.73
Model 4	$I_{i}=\alpha +\hat{\theta }\times F_{i}$	0.172	916.26	438.5	331.42–545.69
Model 5	*I*_*i*_ = *α* + *θ*_1_ × *F*_*i*_ + *θ*_2_ × *S*_*i*_	0.523	811.49	438.5	324.86–552.24

All models showed a significant p-value < 0.01.

* Proposed model, which has the highest r-squared and the lowest AIC.

** Model 2, which is the model without intercept, is the only model that cannot estimate the national incidence.

**Fig 4:**
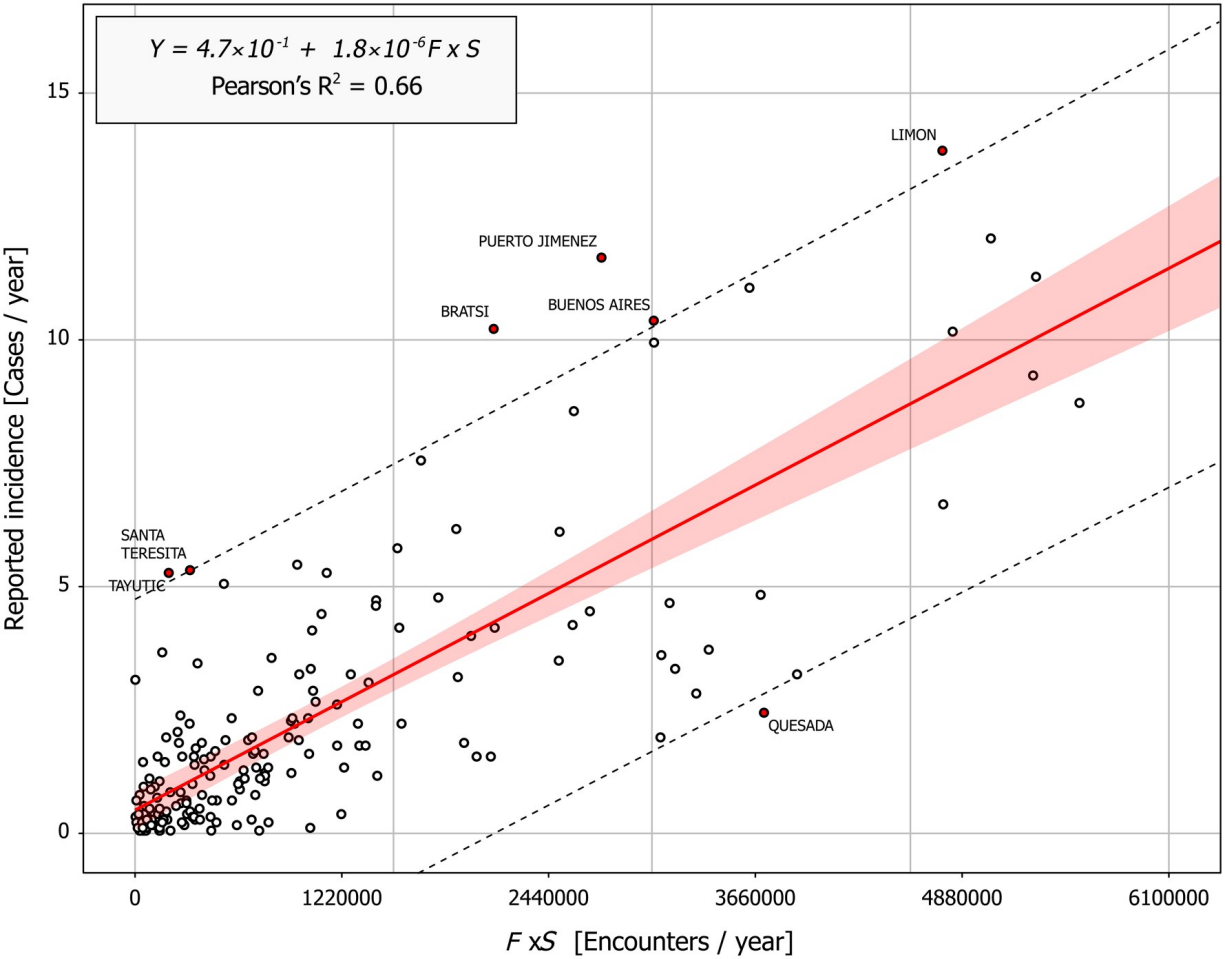
Linear regression for proposed model and reported incidence. Y-axis corresponds to reported incidence per each district, and x-axis is our estimation of the frequency of encounters with the study species times the rural population for each district. Each point represents the reported incidence and our estimation for each district. The red line indicates the predicted regression, and red shading indicates the 99% confidence interval. The dotted line is the prediction interval. The lineal behavior corresponds with the assumptions of the model, and we found a positive and significant correlation between both variables. Labeled districts are those in which the prediction fell outside of the prediction interval. Figure was produced by using ggplot package in r environment.

The overall snakebite incidence estimated by model 1 was 438.5 (ranging from 361.29 to 515.82) cases per year. We found a significant association (r-squared = 0.66, p<0.01) between the reported snakebite incidence and predicted venomous snake encounters (*F*_*i*_ × *S*_*i*_, where *F*_*i*_ is the district averaged encounter frequency with *B*. *asper* and *S*_*i*_ is the rural population per district) (Fig 4). Since the residuals of this model did not vary across fitted values (p>0.01 for the Pearson’s product moment correlation test), the linear regression met the assumptions of normality and heteroscedasticity.

The prediction interval for the model was ± 4.41 cases. Seven districts (4% of the total used districts) (Fig 4) exhibited deviations between predicted and reported incidence that were greater than this interval, six underestimated by the model (Puerto Jiménez, Bratsi, Santa Teresita, Buenos Aires, Limón, and Tayutic), and one overestimated by it (Ciudad Quesada).

## Discussion

Our model successfully captures and estimates snakebite incidence. This is the simplest model that can be performed by using the law of mass action, which is the basis for the development of more complex models [15]. These complex models can estimate relevant variables in public health such as rate of patients recovery, mortality, and disability [15,16,19]. Such approaches have been successfully used for other Neglected Tropical Diseases, including diseases with more complicated transmission dynamics than snakebite [39,40]. Thus, our model constitutes a base model to understand snakebite epidemiology, and it could be expanded to estimate mortality, morbidity, and economic burden to ultimately optimize medical responses to snakebite [41,42]. Our model accurately estimated the national incidence of snakebite in Costa Rica and its range (Model prediction: 438.5 ± 77.2 yearly cases; Reported incidence: 438.5 yearly cases, minimum: 359 in 1991, maximum: 518 in 2006), captured 66% of the variation of district data, and had a significant r-squared value similar to that reported for more complex spatial regressions of other health problems (range 0.54–0.7) [43,44]. Therefore, our model suggests that the spatial distribution of snakebite is mostly driven by a combination of environmental factors affecting venomous snakes’ distribution and their association with exposed human population size.

The distribution of venomous snakes has been used as an input to determine the effect of climate change on snakebite burden [45], and to determine vulnerable human populations [46]. Our algorithm uses those distributions to understand the geographical variation of snakebite, but more importantly we can estimate incidence in places where data is scarce. The fact that the law of mass action helps to account for snakebite spatial heterogeneity in our model, confirms that incidence is directly affected by abundance of venomous snakes. Our results underscore the importance of incorporating the abundance—and not the number—of medically important snake species as a risk estimator. Recently, studies oriented to identify vulnerable populations using diversity of dangerous venomous snakes as risk index do not highlight Latin America as vulnerable [46]. We suggest that Latin America has several vulnerable populations related to fewer but more abundant venomous snakes’ species.

### Mathematical model performance

Our species distribution model adequately predicts the distribution of *B*. *asper* in Costa Rica, as high AUC values (>0.75) indicate a reliable distribution estimation [47]. Our estimated distribution agrees with previous studies in which the distribution range of *B*. *asper* was estimated based on presence records at natural history museums [26,48]. Additionally, our encounter frequency estimations of *B*. *asper* across an altitudinal gradient corroborate previous studies that suggest that *B*. *asper* prefers the lowlands [21,48]. The highest encounter frequency was in the humid Pacific lowlands during the dry season, close to water bodies. Because we did not measure population sizes, we cannot evaluate whether differences in encounter frequency between the Caribbean and Pacific lowlands are attributed to demographic disparities. We hypothesize that the marked dry season in the Pacific restricts available water bodies, which may motivate *B*. *asper* to aggregate near water, and in turn raise the likelihood of human-snake encounters in the Pacific lowlands [21,49].

The models based on the law of mass action showed the strongest performance predicting snakebite incidence in Costa Rica. In fact, model 1 and model 2 share the greatest r-squared values with the lowest AIC, but only model 1 estimated well the national incidence (View Table 1). Then models based on this law estimates better snakebite incidence than other models based on *F* and *S*. In addition, given that the maximum reported incidence for a district was 15 cases per year, the value for the intercept in the proposed model (Model 1) was clearly close to zero (0.47 cases per year). Then, our proposed model outperformed the other models. The slope for our model, the parameter *θ*, represents the conditional probability that an encounter with a venomous snake ends in a bite. Although this parameter is significantly different from zero, its value is very low (1.8 x 10–6); possibly because our estimation is based on an active search to estimate the venomous snakes encounter frequency. The encounter frequency between an exposed human population and venomous snakes is much smaller, which increases the conditional probability. Despite the low value of *θ*, our model continues to estimate the incidence of snakebite adequately.

In a few districts, estimated and reported incidences did not match. This could be explained by two conditions which are not mutually exclusive. First, some districts may have included reports of snakebite that occurred in neighboring districts. Data collected by the CCSS is based on the attending medical center, rather than on the site of envenomation. For example, Puerto Jiménez, Limón and Buenos Aires are large towns with at least one CCSS health clinic, so patients from surrounding rural districts could be referred to those centers [50]. Second, the model assumes spatial homogeneity in encounter frequency and susceptible human population distribution within each district, which is obviously an approximation. Districts could include areas with elevations where *B*. *asper* is not distributed, but human population can be living in elevations where *B*. *asper* is found: Our model will underestimate incidence in these places. For example, a large portion of Tayutic, Bratsi and Santa Teresita districts is located in highlands where *B*. *asper* is not found, whereas human population is concentrated at lower elevations [50]. This could explain why reported incidence in these districts is higher than estimated incidence. Conversely, overestimations might reflect a bias due to spatial heterogeneity of the human population. For example, the 36% of the population of Quesada are catalogued as rural, but Quesada contains the biggest city in northern Costa Rica [51]. *B*. *asper* is distributed across this district, but most rural people work in urban areas and are not prone to encounters, leading to a higher estimated incidence than the reported by medical centers [52].

Our model has the potential to estimate snakebite incidence in places where data is not available despite the few mismatches discussed previously. This model estimated incidence adequately by only using presence records for venomous snakes, measurements of encounter frequency with venomous snakes, and census data for rural human populations. This methodology is simpler than performing community-based studies, so by using this model a rapid and affordable estimation for snakebite incidence can be done. This model could be used for other species of venomous snakes once encounter frequency surveys are performed. Additionally, this model can be improved: Incorporating spatial data on population density will resolve issues with rural/urban heterogeneity, as well as including map layers of socioeconomic factors will highlight particularly at-risk populations. Given that the estimation of snakebite burden has been flagged as a critical goal to improve public health policy, the proposed model can play a crucial role in the planning and management done to minimize snakebite burden in areas where epidemiological data is scarce [7,8].

### Limitations and challenges

Although our model performed well at estimating snakebite incidence, our methodology exhibits different challenges: 1) It requires reliable occurrence data, which is not always available for tropical venomous snake species [53,54]. 2) A baseline incidence dataset is needed to calibrate and train the model, so its application in countries where snakebite incidence is poorly reported may be challenging. 3) Our methodology relies on the estimation of the encounter frequency (*F*). This estimation requires a high field work effort and qualified personnel in searching venomous snakes.

In the presence of the conditions described above, our model has the potential to be used in other regions with snakebite envenomation. Distribution of venomous snakes can be estimated by using data for other countries, and the model could be calibrated by using a subset of districts with reliable data, or using snakebite data for neighbor countries. Such extrapolations must be done carefully, because snakes’ distribution could change drastically between regions; for example in Costa Rica *B*. *asper* reaches a maximum of 1200 m.a.s.l. [26], but in Ecuador it is found up to 1900 m.a.s.l. [55].

Finally, to use the model in places where snakebite is caused by multiple species, such as Colombia, where *Bothrops asper* and *Bothrops atrox* cause the majority of envenomations, we recommend to measure encounter frequency for each species separately. Then, to calibrate the model it would be necessary to perform a multiple lineal regression since the parameter θ may vary between species. This parameter represents the probability that an encounter with a snake species ends in a bite. The model accounting for multiple species would be: $I_{i}=\alpha +\sum_{j=1}^{N}\left(\theta_{j}\times F_{j,i}\times S_{i}\right)$, where *j* is the sub-index for each snake species, *F*_*j*,*i*_ × *S*_*i*_ are the independent variables, and *I*_*i*_ is the response variable.

In summary, our study highlights the effectiveness of mathematical models estimating snakebite incidence and the importance of including venomous snakes’ biology to understand snakebite epidemiology. The lack of this biological information currently becomes a challenge to fit this kind of models. Therefore, it is important to move forward in the knowledge of the biology and natural history of venomous snakes (distribution, population dynamics, habitat use, etc.) [21,54]. Even though this model can estimate incidence, it will never substitute a proper epidemiological surveillance program. Such programs must be adopted by public health authorities in countries affected by snakebite to minimize impact in at-risk populations, and to eliminate the neglect of snakebite as a tropical disease [8].

### Data availability

The data that support the findings of this study are openly available in figshare at https://doi.org/10.6084/m9.figshare.8311994 [56].

## Supporting information

S1 Fig Presence locations for *Bothrops asper*. Map was produced by using QGIS Geographic Information System, Open Source Geospatial Foundation Project. http://qgis.osgeo.org.
(TIF)

S2 Fig **(a) Rural population by district. (b) Estimated average encounter frequency by district**. Maps were produced by using QGIS Geographic Information System, Open Source Geospatial Foundation Project. http://qgis.osgeo.org.
(TIF)

S3 Fig **(a) Reported incidence by district. (b) Estimated incidence by district**. Maps were produced by using QGIS Geographic Information System, Open Source Geospatial Foundation Project. http://qgis.osgeo.org.
(TIF)

S4 Fig Analysis of omission/commission for the maximum entropy model. Note that the omission on training and test samples is close to the predicted omission, so the model fulfills the assumptions of maximum entropy. Figure was produced by using r environment.
(TIF)

S5 Fig Receiver operating characteristic (ROC) curve for the maximum entropy model. Figure was produced by using r environment.
(TIF)
