# Supplementary figures and images for: Estimating snakebite incidence from mathematical models: A test in Costa Rica
Source: PLoS Negl Trop Dis. 2019 Dec 2;13(12):e0007914. doi: 10.1371/journal.pntd.0007914 (PMC6907855; doi:10.1371/journal.pntd.0007914)

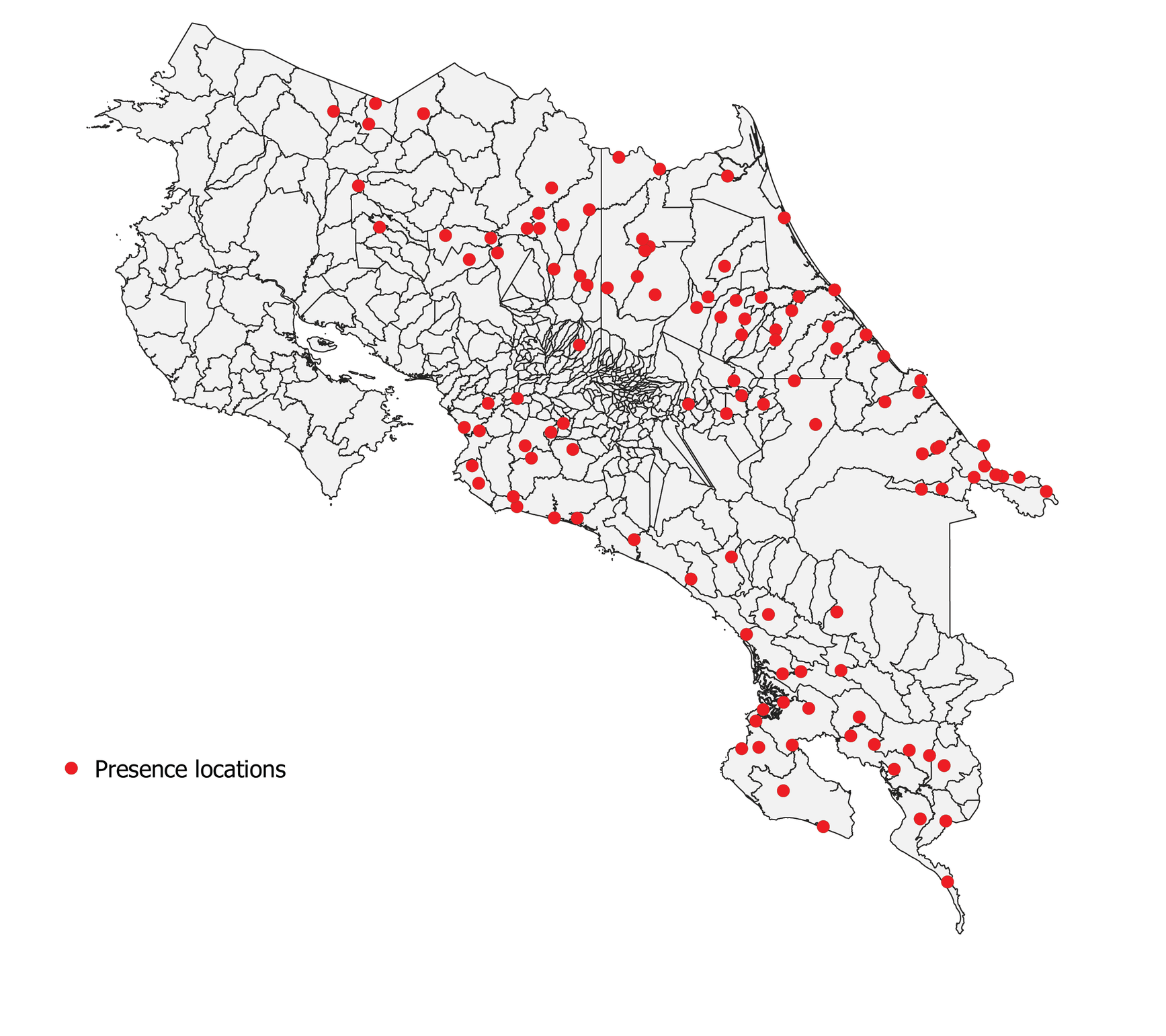

Supplement: S1 Fig — Map was produced by using QGIS Geographic Information System, Open Source Geospatial Foundation Project. http://qgis.osgeo.org. (TIF) [file pntd.0007914.s001.tif]

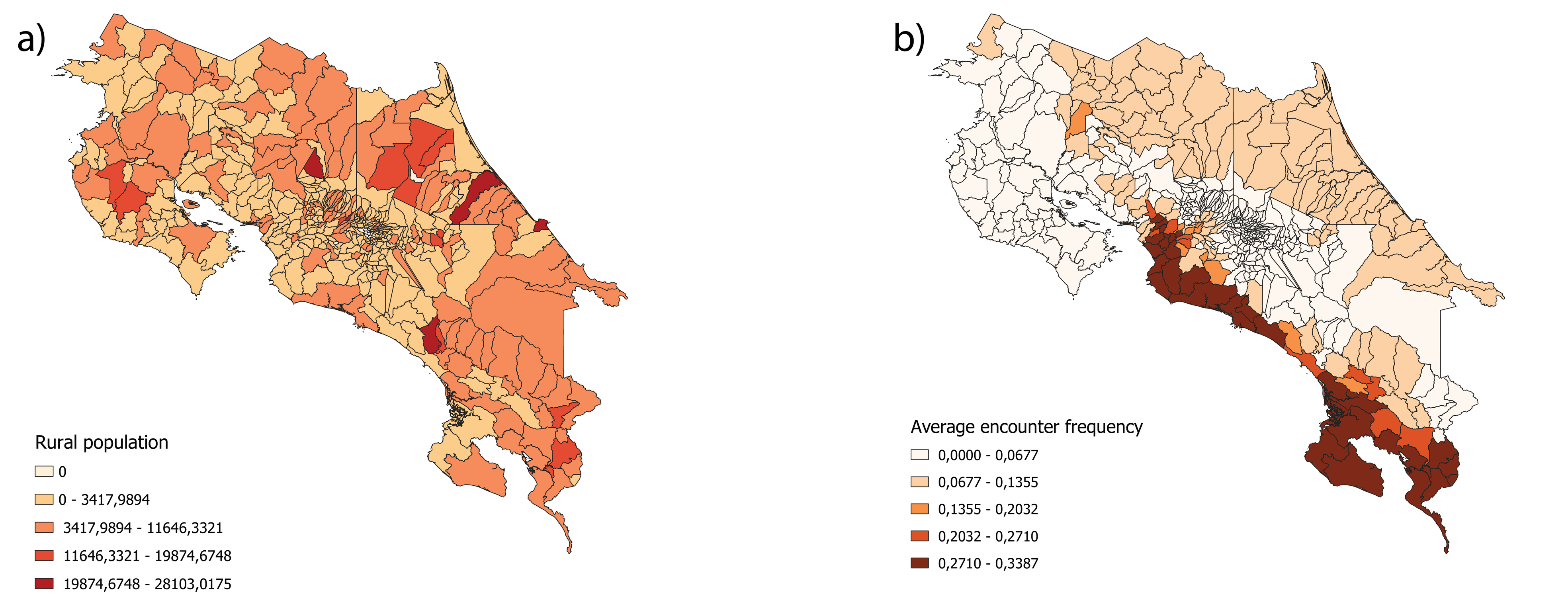

Supplement: S2 Fig — (a) Rural population by district. (b) Estimated average encounter frequency by district. Maps were produced by using QGIS Geographic Information System, Open Source Geospatial Foundation Project. http://qgis.osgeo.org. (TIF) [file pntd.0007914.s002.tif]

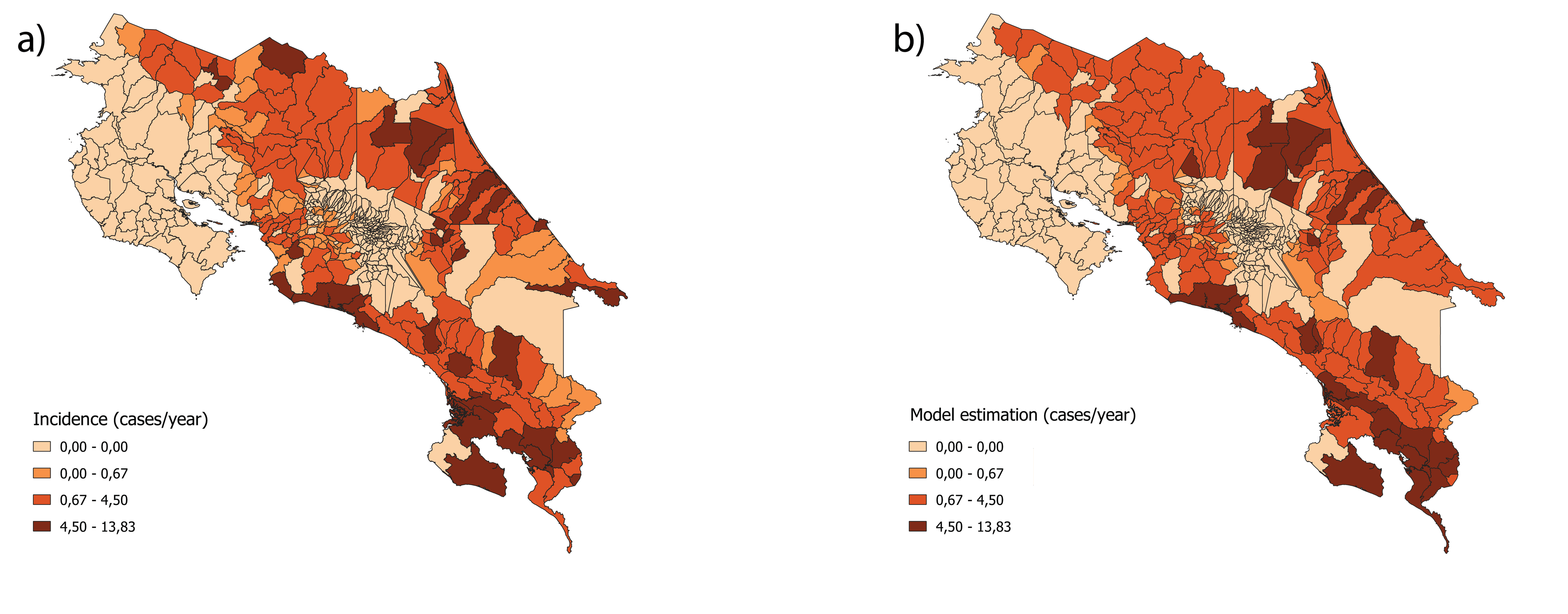

Supplement: S3 Fig — (a) Reported incidence by district. (b) Estimated incidence by district. Maps were produced by using QGIS Geographic Information System, Open Source Geospatial Foundation Project. http://qgis.osgeo.org. (TIF) [file pntd.0007914.s003.tif]

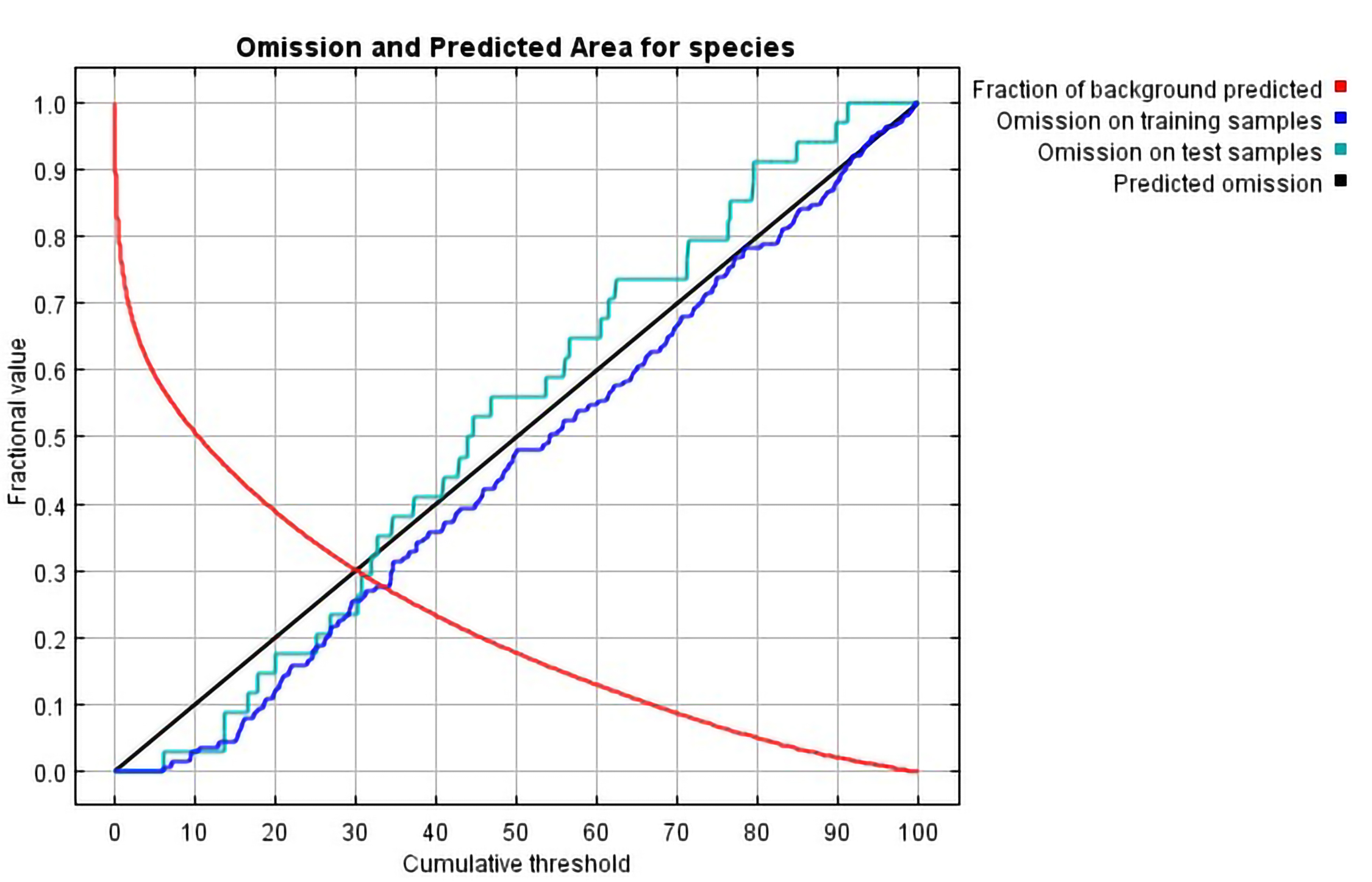

Supplement: S4 Fig — Note that the omission on training and test samples is close to the predicted omission, so the model fulfills the assumptions of maximum entropy. Figure was produced by using r environment. (TIF) [file pntd.0007914.s004.tif]

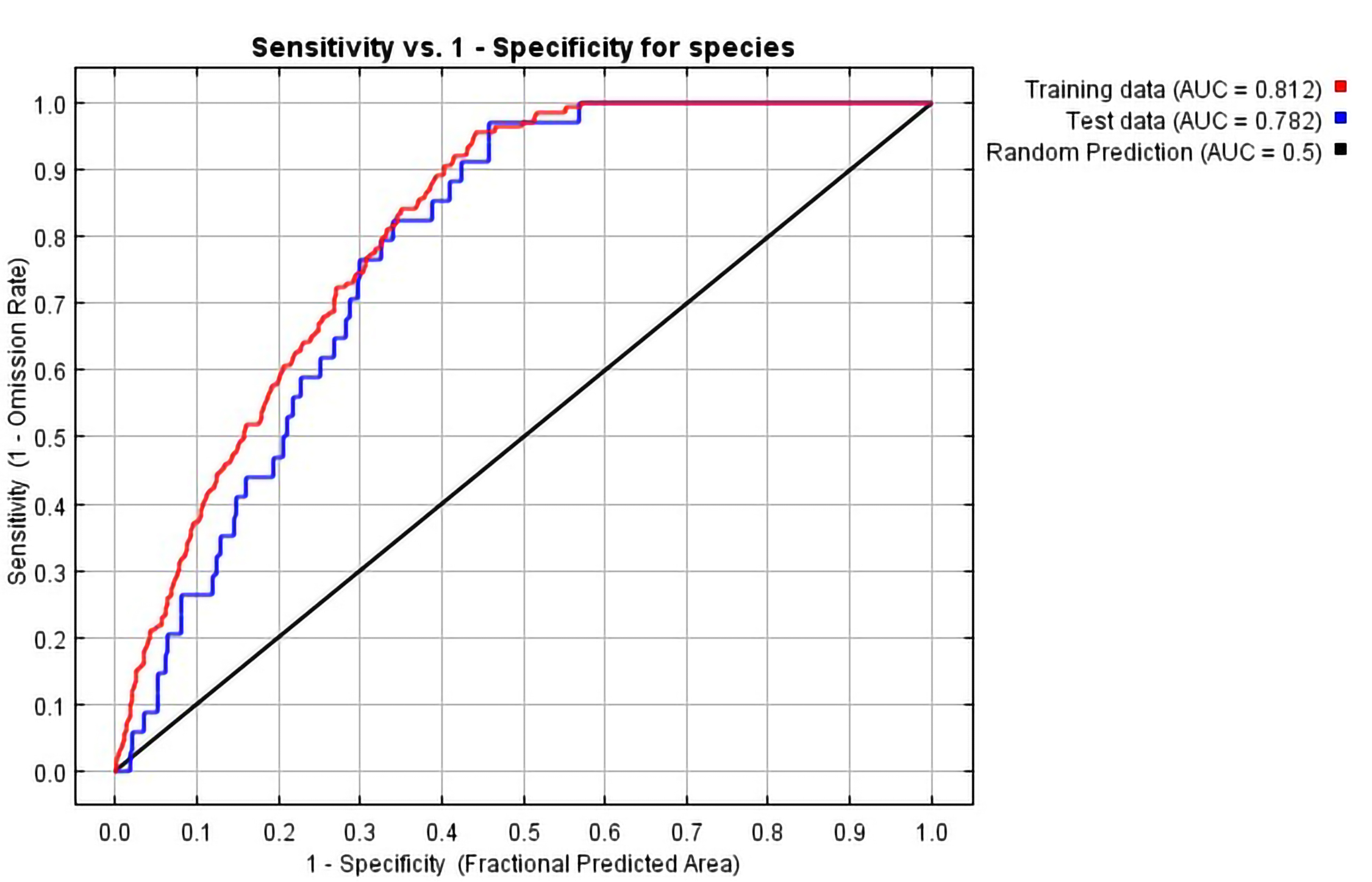

Supplement: S5 Fig — Figure was produced by using r environment. (TIF) [file pntd.0007914.s005.tif]
